# Heart of Lymphoma: Primary Mediastinal Large B-Cell Lymphoma with Endomyocardial Involvement

**DOI:** 10.1155/2013/814291

**Published:** 2013-10-01

**Authors:** Elisa Rogowitz, Hani M. Babiker, Ravitharan Krishnadasan, Clint Jokerst, Thomas P. Miller, Michael Bookman

**Affiliations:** ^1^University of Arizona College of Medicine, 1501 N. Campbell Avenue, Tucson, AZ 85724, USA; ^2^Division of Hematology-Oncology, University of Arizona Cancer Center, 1515 N. Campbell Avenue, Tucson, AZ 85724, USA; ^3^Department of Radiology, University of Arizona College of Medicine, 1501 N. Campbell Avenue, Tucson, AZ 85724, USA

## Abstract

Primary mediastinal B-cell lymphoma (PMBCL) is an uncommon aggressive subset of diffuse large B-cell lymphomas. Although PMBCL frequently spreads locally from the thymus into the pleura or pericardium, it rarely invades directly through the heart. Herein, we report a case of a young Mexican female diagnosed with PMBCL with clear infiltration of lymphoma through the cardiac wall and into the right atrium and tricuspid valve leading to tricuspid regurgitation. This was demonstrated by cardiac MRI and transthoracic echocardiogram. In addition, cardiac MRI and CT scan of the chest revealed the large mediastinal mass completely surrounding and eroding into the superior vena cava (SVC) wall causing a collar of stokes. The cardiac and SVC infiltration created a significant therapeutic challenge as lymphomas are very responsive to chemotherapy, and treatment could potentially lead to vascular wall rupture and hemorrhage. Despite the lack of conclusive data on chemotherapy-induced hemodynamic compromise in such scenarios, her progressive severe SVC syndrome and respiratory distress necessitated urgent intervention. In addition to the unique presentation of this rare lymphoma, our case report highlights the safety of R-CHOP treatment.

## 1. Case Report

A 23-year-old Mexican female presented to the emergency room with a relentless cough for three days. The cough and dyspnea started six weeks prior to presentation and were gradually worsening, causing her to sleep upright. She also experienced fatigue, prominent facial swelling, engorged neck vasculature, headaches, and a 25-pound weight loss. Climbing even four individual stairs caused this former soccer athlete significant fatigue and lightheadedness. She denied having fevers, chills, or night sweats. She is a full-time college student living with her parents and sibling in Mexico. She was evaluated there and diagnosed with Cushing syndrome and hypothyroidism and was prescribed levothyroxine. She presented to our emergency department after her condition deteriorated during her visit to the USA.

The patient had a temperature of 37.2°C, a pulse of 120 beats per minute, a blood pressure of  96/57 mm Hg, a respiratory rate of 24 breaths per minute, and an oxygen saturation of 98% on room air. She had significant facial, neck, and upper trunk swelling with visible engorged vessels. A collar of stokes was present, and her right upper extremity was more edematous than the left. A faint diastolic murmur was heard best over the right sternal border. No lymphadenopathy was noted. Labs revealed a WBC of 11.4 (3.4–10.4 1000/uL) with 81% neutrophils, 11% lymphocytes, 6% monocytes, 1% eosinophils, 1% basophils, and an absolute neutrophil count of 9234/microL. Serum LDH was 1308 (125–243 IU/L). In addition, her potassium was 3.2 (3.5–5.1 mMol/L), calcium 9.8 (8.6–10.6 mg/dL), phosphorus 4.2 (2.3–4.7 mg/dL), and magnesium 2 (1.6–2.6 mg/dL). Beta-2-microglobulin was 1.82 (0.97–2.64 mg/L), and uric acid was 4.4 (2.6–6 mg/dL).

**Figure 1:**
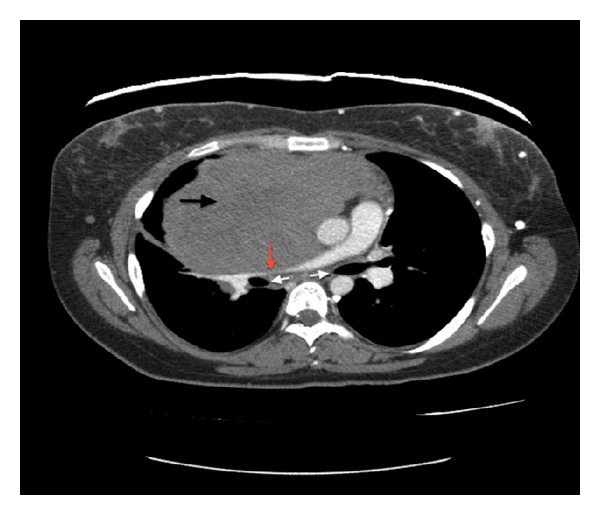
CT scan of the chest with contrast reveals a large lobulated anterior mediastinal solid mass (black arrow) with extension into the right hemithorax and the right atrium. There is displacement of the great vessels into the left hemithorax with significant mass effect on the right upper lobe. The tumor causes compression of the right pulmonary artery (red arrow) and right and left mainstem bronchi (white arrows).

**Figure 2:**
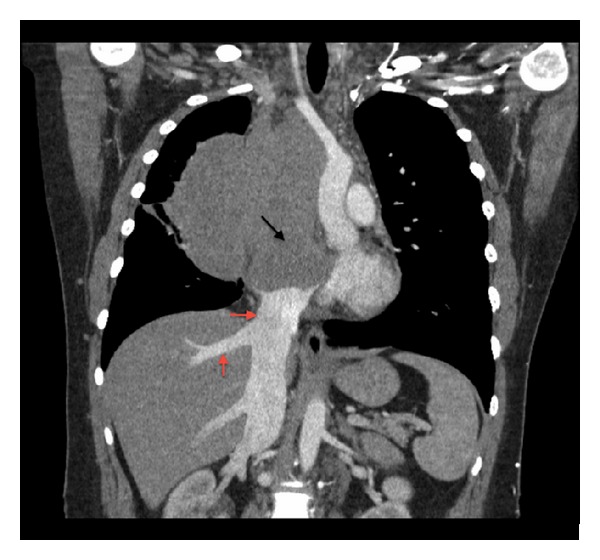
Coronal CT scan image elucidates a mediastinal mass with extension into the right atrium (black arrow) with complete encasement and compression of the SVC. The tumor extends to the confluence of the IVC in the right atrium causing dilatation of the intraabdominal IVC and hepatic veins suggesting compromised cardiac return (red arrows). Tumor causes the displacement of great vessels into the left hemithorax.

**Figure 3:**
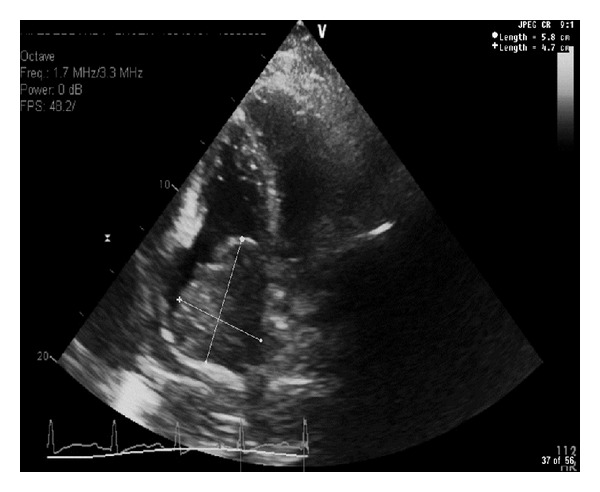
A comprehensive 2 dimensional M-mode color flow and Doppler echocardiography reveals a normal left ventricular systolic function (EF 60–69%). A large right atrial mass measuring 5.8 × 4.7 cm almost fills the right atrium and extends into the tricuspid valve causing tricuspid regurgitation.

**Figure 4:**
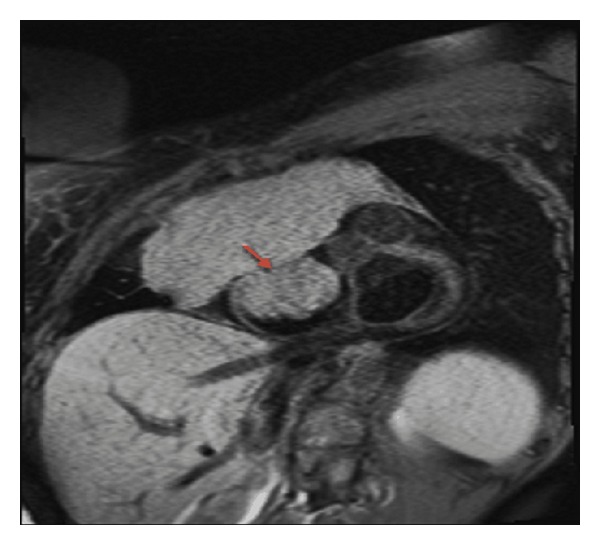
Cardiac MRI short axis T1 at the level of mitral valve reveals a large mediastinal mass infiltrating and obliterating the SVC causing SVC obstruction. The tumor extends into the right atrium (red arrow) and invades the tricuspid valve. Maximum diameter of the mediastinal mass measures 15 × 10 cm, and the mass in the right atrium measures 5.8 × 5.3 cm.

A chest X-ray demonstrated a large anterior mediastinal mass. Follow-up contrast enhanced chest CT revealed a large lobulated anterior mediastinal mass near the right atrium with complete encasement and effacement of the superior vena cava (SVC) and invasion into the right atrium (Figures 1 and 2). Tumor almost completely filled the right atrium resulting in significant dilation of the inferior vena cava, hepatic veins, and portal vein. CT imaging also revealed superior and anterior mediastinal lymphadenopathy. A transthoracic echocardiogram demonstrated a mass with erosion through the SVC and extension through the endocardium and into the right atrium up to the tricuspid valve annulus resulting in regurgitation (Figure 3). The ejection fraction was normal at 60–69%. An MRI defined a 15 × 10 cm anterior mediastinal mass infiltrating through the myocardium into the right atrial lumen, associated with complete SVC obstruction (Figure 4). 

A CT-guided biopsy of the mass revealed atypical large lymphoid cells with prominent nucleoli, abundant cytoplasm, and nodular fibrosis. The neoplastic cells had positive expression of CD20 (diffuse), CD23, CD45, Bcl-6, Mum1, Pax-5, CD30 (dim), Bcl-2 (dim), and HLA-DR (dim). The cells were negative for ALK-1, CD10, CD19, and surface immunoglobulins. Bone marrow biopsy, aspiration, and cytogenetics were normal. 

## 2. Discussion

The presentation of this 23-year-old female with progressive airway compromise and SVC syndrome correlated with the imaging findings. The bulky mediastinal mass caused progressive tracheal and bronchial compression and irritation that resulted in her cough and inability to lay supine. The SVC obstruction was consistent with the prominent vascularity and edema of her face and upper trunk. The swelling was so marked that it was initially interpreted as Cushingoid.

In a young female, a large anterior mediastinal mass is most likely lymphoma, thymoma, germ cell tumor, or sarcoma. Mediastinal lymphoma could represent nodular sclerosis subtype of classical Hodgkin's lymphoma (NScHL) or less commonly, a primary mediastinal B cell lymphoma. Based on the morphologic and immunophenotypic features of the biopsy, the final diagnosis was consistent with PMBCL. The tissue was positive for the expression of B-cell antigen CD20, dim CD30, negative surface immunoglobulins, and morphologically lacking Reed-Sternberg cells, hence excluding NScHL as the diagnosis. The stage was determined to be II-EA by the Ann-Arbor Staging criteria.

PMBCL is an uncommon clinic-pathological entity recognized by the WHO classification of lymphoid malignancies [1]. It represents 2-3% of non-Hodgkin's lymphomas and has a propensity to affect young women in their third and fourth decades [1]. PMBCL arises from putative thymic medullary B-cells [2]. Many cases have fine compartmentalizing sclerosis and rarely Reed-Sternberg cells [3]. Flow cytometry to determine the immunophenotype of the tumor cells typically demonstrates the expression of B-cell associated antigens (CD19, CD20, and CD22), CD45, and CD30 (weak) and an absence of surface immunoglobulins, CD5, and CD10 [2]. TRAF-1 and nuclear c-Rel staining, a characteristic finding in Reed-Sternberg cells, are also often positive [2]. The lack of immunoglobulin expression at the protein, mRNA level, and frequent CD30 expression, although dim in PMBCL, is typical of PMBCL and classical Hodgkin's Lymphoma (cHL) [3]. This pattern is uncommon in other forms of diffuse large B-cell lymphoma (DLBCL). Despite the shared commonalities with cHL, PMBCL is considered a subset of DLBCL because of the B-cell program conservation and Bcl-6 protein synthesis (contrary to the silencing in cHL) [3, 4]. Cytogenetically, PMBCL may harbor chromosomal aberrations including gains in chromosome 9p and 2p [5].

Our patient's presentation is typical of PMBCL. It is nearly always associated with SVC syndrome or airway compression [3]. 75% of patients present with a mass greater than 10 cm [2, 3, 6]. About 50% of patients have systemic B signs (fever, night sweats, and weight loss), pleural, or pericardial effusion, and 77% have elevated LDH [3]. Some patients have local extension into the lung, chest wall, pleura, or pericardium [2, 3, 6]. Bone marrow or distant lymph node involvement at presentation is unusual [4]. 

This particular patient's case was interesting because of the clear infiltration through the cardiac wall and invasion of the right atrium leading to tricuspid regurgitation. Cardiac MRI and echocardiogram demonstrated the mass infiltrating through the pericardium, myocardium, and endocardium and terminating in the right atrial lumen as a tennis ball-sized mass. Our patient also had complete encirclement and invasion through the SVC wall. SVC and cardiac infiltration created a significant therapeutic challenge because lymphomas are very responsive to chemotherapy and treatment can lead to vascular wall rupture and hemorrhage. However, her progressive severe SVC syndrome and respiratory distress necessitated urgent intervention. Following the diagnostic biopsy, respiratory distress was initially managed with systemic steroids, which rapidly improved her symptoms, and she could sleep flat for the first time in many weeks. This was consistent with the known sensitivity of lymphoma to steroid therapy. Following pathologic confirmation of PMBCL, the decision was made to treat with R-CHOP therapy but with omission of rituximab during her first cycle to minimize the risk of myocardial rupture. The first cycle was well tolerated without tumor lysis syndrome, hemodynamic compromise, or other acute toxicities. She continued her treatment in Mexico and has currently completed cycle two through four with R-CHOP therapy without treatment related toxicities. Repeat imaging and echocardiography showed the resolution of the right atrial mass and mediastinal mass regression. Her SVC and airway symptoms resolved. 

Our case report highlights the safety of CHOP in such scenario with close monitoring of the patient's vitals. In addition, it highlights epidemiology, diagnosis, pathology, and treatment of this rare lymphoma. 

The optimal chemotherapy role and consolidative radiotherapy in patients with PMBCL are not well defined. The current standard of treatment is rituximab, cyclophosphamide, doxorubicin, vincristine, and prednisone (R-CHOP) [7]. A recent single-group, phase 2, prospective study using dose-adjusted etoposide, doxorubicin, and cyclophosphamide with vincristine, prednisone, and rituximab (DA-EPOCH-R) and filgrastim without radiotherapy in 51 patients revealed a high event free survival of 93% and an overall survival of 97% over 5 years alluding to a role for this regimen [8]. Studies by the Southwest Oncology Group comparing CHOP to 2nd and 3rd generations chemotherapy in the treatment of DLBCL occurred prior to the recognition of PMBCL as a distinct entity. Many studies were conducted in the prerituximab era. Other studies indicated the superiority of intensified-dose chemotherapy such as methotrexate, cyclophosphamide, doxorubicin, vincristine, prednisone, and bleomycin (MACOP-B) [6]. However, some of these were based on historical data without randomized comparison and could be subject to selection bias and low power. Many patients received consolidative radiotherapy, although the progression free and overall survival is not adequately delineated with some studies revealing no benefit [8, 9].
